# An unexpected sequel to happiness: happy heart syndrome

**DOI:** 10.1093/omcr/omag108

**Published:** 2026-07-08

**Authors:** Laith Rhabneh, Jamil Wafi, Ra’ed Ababneh, Shahd Qaddour, Mohammed Aloqaily, Abdel Rahman Alwardat, Khalid Hazaimeh

**Affiliations:** Department of Internal Medicine, Hackensack Meridian Ocean University Medical Center, Brick Township, NJ 08724, United States; Department of Internal Medicine, Hamad Medical Corporation, Doha, Qatar; Department of Internal Medicine, Hackensack Meridian Ocean University Medical Center, Brick Township, NJ 08724, United States; Diagnostic Radiology Department, King Abdullah University Hospital, Irbid, 22110, Jordan; Internal Medicine Department, University of Maryland Midtown Campus, Baltimore, MD 21201, United States; Department of Internal Medicine, King Abdullah University Hospital, Ramtha, Irbid, 22110, Jordan; Jordan Ministry of Health, Amman, 11118, Jordan

**Keywords:** cardiovascular, Takotsubo syndrome, acute coronary syndrome mimic

## Abstract

Takotsubo cardiomyopathy (TCM) is characterized by transient regional systolic dysfunction without obstructive coronary artery disease. A rare subtype, happy heart syndrome (HHS), is triggered by positive emotional events. A 65-year-old woman with hypertension, diabetes mellitus, and dyslipidemia presented with typical anginal chest pain for three days, beginning after attending a wedding party. Initial clinic ECG showed anterolateral ST-segment elevation, prompting referral to the Emergency Department. On arrival, her chest pain had resolved, and repeated ECG demonstrated normalization of ST changes with QT prolongation and T-wave inversion. Troponin was elevated at 717 ng/l. Echocardiography showed an EF of 35% (baseline 58%) with apical and LAD-territory akinesia and ballooning. Coronary angiography revealed normal coronaries, while left ventricular angiography demonstrated findings consistent with TCM. Cardiac MRI excluded infarction or fibrosis. With guideline-directed therapy, EF recovered to 56% on follow-up.

## Introduction

Takotsubo syndrome (TTS), also known as stress cardiomyopathy or ‘broken heart syndrome,’ is characterized by transient left ventricular systolic dysfunction in the absence of obstructive coronary artery disease [1]. The condition often mimics acute coronary syndrome both clinically and electrocardiographically, with symptoms such as chest pain, ST-segment changes, and elevated cardiac biomarkers [2].

Although TTS is widely recognized in clinical practice, it remains relatively rare, with an estimated incidence ranging between 1%–2% of all suspected acute coronary syndrome presentations [3]. Interestingly, a lesser-known and even rarer variant of TTS is happy heart syndrome (HHS), wherein the inciting trigger is a positive or joyful emotional event rather than a negative or distressing one [4]. This form was first described by Ghadri et al. in 2016, who highlighted that intense positive emotions can precipitate similar transient cardiac dysfunction, challenging the traditional view of TTS as solely a ‘broken heart’ phenomenon [5].

Here, we present a case illustrating this exceptionally rare and diagnostically challenging entity. It involves a middle-aged woman who experienced extreme joy at her daughter’s wedding. Unfortunately, this overwhelming happiness was followed days later by the onset of HHS. Despite the initially alarming presentation, she achieved complete cardiac recovery and continues to live healthily, looking forward to witnessing the happiest milestones of her other children in the years ahead.

## Case history/examination

The patient is a 65-year-old lady with a previous medical history including hypertension, type II diabetes mellitus, and dyslipidemia. She had been following in cardiology outpatient clinics for atypical chest pain for several months prior to her presentation. Outpatient workup of electrocardiogram (ECG) and transthoracic echocardiography turned out to be normal. The patient was hesitant to continue the rest of the diagnostic tests and lost follow up.

Months later, She sought medical care for typical anginal chest pain for 3 days. The pain was associated with shortness of breath that becomes more prominent with physical activity. There was no lower limb edema, orthopnea or paroxysmal exertional dyspnea.

## Differential diagnosis, investigations and treatment

As part of standard care for chest pain, ECG, troponin, and the rest of the blood workup were ordered. She was found to have ST segment elevation (STE) in the anterolateral leads (Fig. 1). She was urgently referred to the emergency department of a nearby tertiary hospital where a follow-up ECG showed resolution of the STE (Fig. 2) and disappearance of the symptoms. She was treated as a case of ACS and admitted to the Cardiac Intensive Care Unit, SCAI A classification. Coronary angiography was done urgently, and the results were surprisingly normal (Fig. 3). However, left ventricular angiogram revealed apical ballooning, which is suggestive of Takotsubo Cardiomyopathy (Fig. 4).

**Figure 1:**
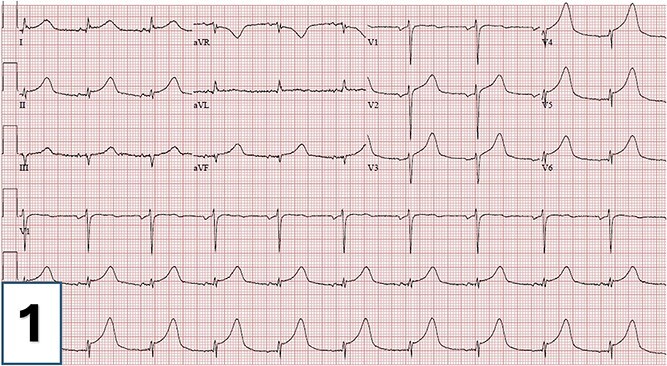
Electrocardiogram (ECG) showing initial ST-segment elevation in the anterolateral leads during presentation at the primary health care corporation.

**Figure 2:**
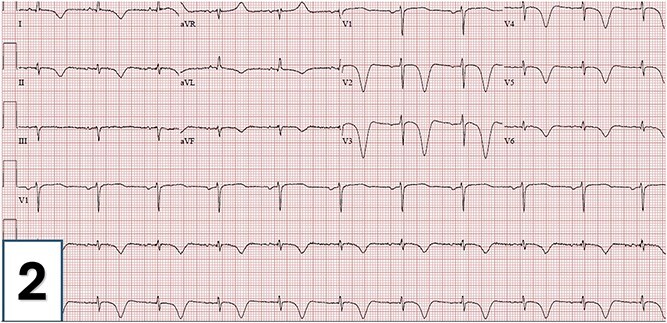
Electrocardiogram (ECG) showing resolution of ST-segment elevation with prolonged QT interval and T wave inversion, recorded upon arrival at the emergency department.

**Figure 3:**
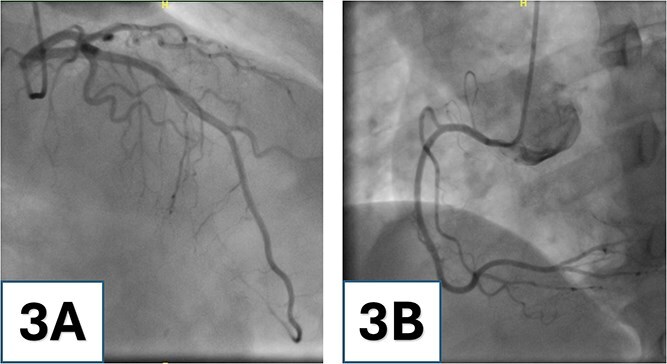
Coronary angiography demonstrating normal epicardial coronary arteries. (A) Left coronary angiogram obtained in the left anterior oblique (LAO) cranial projection. (B) Right coronary angiogram obtained in the left anterior oblique (LAO) projection.

**Figure 4:**
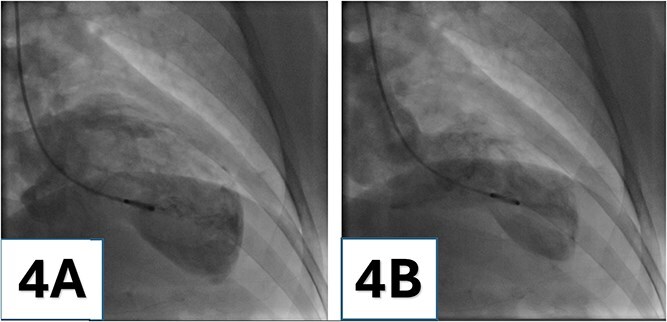
Left ventriculogram demonstrating apical ballooning characteristic of Takotsubo cardiomyopathy, showing the end-diastolic phase (4A) and end-systolic phase (4B).

For further evaluation, the treating team performed transthoracic echocardiography (Fig. 5), which confirmed moderately reduced systolic left ventricular function with a drop in ejection fraction from 58% five months ago to 35%, with apical akinesia and hyperkinetic remaining segments of the left ventricle.

**Figure 5:**
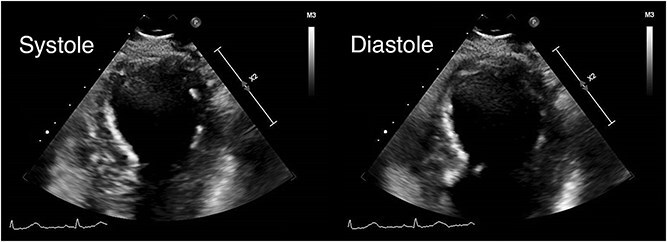
Apical two-chamber view on transthoracic echocardiography showing left ventricular apical ballooning with systolic akinesis of the apical segments and basal hyperkinesis, characteristic of Takotsubo cardiomyopathy.

Laboratory workup during her stay was significant for an elevated troponin of 717 ng/l, which gradually came down within the next few days. Pro-B-type Natriuretic Peptide (pBNP) was elevated at 633 pg/ml initially and started to decrease gradually.

The diagnosis of Takotsubo Cardiomyopathy was made based on the Mayo Clinic Criteria [6], which includes:

1. Transient left ventricular dysfunction extending beyond a single coronary artery distribution (evident as apical akinesia with hyperkinesis of the remaining segments on echocardiography and angiography).
2. Absence of obstructive coronary artery disease, confirmed by normal coronary angiography findings.
3. New ECG changes (transient ST segment elevation) along with modest troponin elevation (717 ng/l in this case).
4. Absence of pheochromocytoma and myocarditis

Cardiac magnetic resonance imaging (MRI) performed to exclude other potential causes revealed an akinetic left ventricular (LV) apex with mild apical ballooning in some views. There was no evidence of myocardial edema, infarction, or fibrosis. These findings are consistent with Takotsubo cardiomyopathy.

## Outcome and follow-up

The patient was discharged home with optimized medical therapy and scheduled follow-up in the cardiology heart failure clinic. She was able to resume her usual daily activities without any limitations or concerns. Follow-up transthoracic echocardiography demonstrated significant improvement in left ventricular function, with an ejection fraction of 56% (Video 1).

## Discussion

Takotsubo syndrome (TTS) is a reversible condition characterized by reversible dysfunction of the left ventricular systolic function. At presentation, TTS can closely mimic acute myocardial infarction, making diagnosis a bit challenging. It can be triggered by intense emotional experiences, whether negative or positive, which are commonly termed "broken-heart syndrome" (BHS) and "happy-heart syndrome" (HHS), respectively [3, 4].

The first documented case of TTS was reported in 1983 at Hiroshima City Hospital. Since the first case of TTS was reported in 1983, its recognition has grown globally, though it remains underdiagnosed and frequently misidentified. Research suggests that approximately 1%–3% of patients initially suspected of having non-ST-elevation myocardial infarction (NSTEMI) may actually have TTS [2, 7]. Among these cases, the occurrence of HHS is particularly rare, accounting for about 4.1% of TTS cases, having the odd association with positive emotional triggers [2].

Takotsubo syndrome (TTS) is a reversible cardiac condition characterized by transient dysfunction of left ventricular systolic function. Clinically, it often mimics acute myocardial infarction, making diagnosis challenging. TTS is typically triggered by intense emotional or physical stress. Depending on the emotional context, TTS is colloquially referred to as ‘broken-heart syndrome’ (BHS) when caused by negative stress, or ‘happy-heart syndrome’ (HHS) when triggered by intense positive emotions [5].

The first documented case of TTS was reported in 1983 at Hiroshima City Hospital [5]. Since then, global awareness has grown, though the condition remains underdiagnosed and often misclassified. It is estimated that 1%–3% of patients initially suspected of having non-ST-elevation myocardial infarction (NSTEMI) are ultimately diagnosed with TTS [7, 8]. Among these, HHS represents a small but distinct subset, accounting for approximately 4.1% of all TTS cases [5].

While both happy heart syndrome (HHS) and broken heart syndrome (BHS) are subtypes of Takotsubo syndrome (TTS) and share hallmark features—such as transient left ventricular dysfunction and absence of significant coronary obstruction—research suggests subtle differences. BHS is typically triggered by negative emotional or physical stress, whereas HHS follows intense positive emotions such as joyful reunions or celebrations [5]. Both conditions involve sympathetic nervous system activation and a catecholamine surge, but the neurohormonal responses may differ depending on emotional valence [6, 9]. HHS is more frequently associated with mid-ventricular or basal ballooning rather than the classic apical pattern of BHS [5], and may also present with less severe symptoms and better short-term outcomes, though further data are needed [1, 5].

The exact mechanisms underlying Takotsubo Syndrome (TTS) are not fully understood, though several theories have been proposed. These include the potential cardiotoxic effects of elevated catecholamines, disruptions in metabolic function, dysfunction of the coronary microvasculature, and spasms involving the proximal segments of the epicardial coronary arteries [4].

Because the clinical presentation of Takotsubo Syndrome (TTS) often mimics that of acute coronary syndrome, establishing a correct diagnosis is critical to ensure appropriate treatment. The Mayo Clinic Diagnostic Criteria provides a useful framework for this purpose. These criteria include transient left ventricular wall motion abnormalities that extend beyond a single coronary artery territory, absence of obstructive coronary artery disease or acute plaque rupture, new ECG changes or modest troponin elevation, and exclusion of other conditions such as pheochromocytoma and myocarditis. Applying these criteria helps differentiate TTS from myocardial infarction and guides proper management [1].

## Conclusion

Happy Heart Syndrome (HHS) is a rare but significant variant of Takotsubo Syndrome, marked by transient left ventricular dysfunction in response to intense positive emotional experiences. Though well-recognized, TTS can be difficult to diagnose initially due to its clinical similarity to Acute Coronary Syndrome (ACS). This challenge is further compounded by the fact that some patients do not present with negative emotional stressors but rather with uplifting life events—an aspect that may be overlooked by clinicians. Careful application of diagnostic tools, such as the Mayo Clinic Criteria, is essential for distinguishing HHS from ACS and avoiding unnecessary interventions. Our case underscores this uncommon presentation and adds to the limited body of literature documenting patients who experienced profound joy followed by the unexpected onset of stress cardiomyopathy. Maintaining a high index of suspicion, even in patients without conventional cardiovascular risk factors, is crucial. With timely recognition and supportive care, patients typically achieve complete recovery, highlighting the reversible and favorable nature of this unique condition.

## Supplementary Material

Video_1_omag108

## Data Availability

All data and materials related to this case report are available from the corresponding author upon reasonable request.
